# Transcriptome and proteome analysis of *Pinctada margaritifera *calcifying mantle and shell: focus on biomineralization

**DOI:** 10.1186/1471-2164-11-613

**Published:** 2010-11-01

**Authors:** Caroline Joubert, David Piquemal, Benjamin Marie, Laurent Manchon, Fabien Pierrat, Isabelle Zanella-Cléon, Nathalie Cochennec-Laureau, Yannick Gueguen, Caroline Montagnani

**Affiliations:** 1 Ifremer - Laboratoire de Biotechnologie et de Qualité de la Perle, Centre Océanologique du Pacifique - BP 7004, 98719 Taravao, French Polynesia; 2 Skuldtech, 134, rue du Curat - Bat. Amarante, 34090 Montpellier, France; 3 UMR CNRS 5561 Biogéosciences, Université de Bourgogne, 6, bd Gabriel, 21000 Dijon, France; 4 IFR 128 BioSciences Gerland-Lyon Sud; UMR 5086 CNRS; IBCP, Institut de Biologie et Chimie des Protéines, Université de Lyon 1, 7 passage du Vercors, Lyon, F-69367, France; 5 Ifremer, Centre de Nantes- rue de l'Ile d'Yeu - BP 21105, 44311 Nantes cedex 03, France

## Abstract

**Background:**

The shell of the pearl-producing bivalve *Pinctada margaritifera *is composed of an organic cell-free matrix that plays a key role in the dynamic process of biologically-controlled biomineralization. In order to increase genomic resources and identify shell matrix proteins implicated in biomineralization in *P. margaritifera*, high-throughput Expressed Sequence Tag (EST) pyrosequencing was undertaken on the calcifying mantle, combined with a proteomic analysis of the shell.

**Results:**

We report the functional analysis of 276 738 sequences, leading to the constitution of an unprecedented catalog of 82 *P. margaritifera *biomineralization-related mantle protein sequences. Components of the current "chitin-silk fibroin gel-acidic macromolecule" model of biomineralization processes were found, in particular a homolog of a biomineralization protein (Pif-177) recently discovered in *P. fucata*. Among these sequences, we could show the localization of two other biomineralization protein transcripts, *pmarg-aspein *and *pmarg-pearlin*, in two distinct areas of the outer mantle epithelium, suggesting their implication in calcite and aragonite formation. Finally, by combining the EST approach with a proteomic mass spectrometry analysis of proteins isolated from the *P. margaritifera *shell organic matrix, we demonstrated the presence of 30 sequences containing almost all of the shell proteins that have been previously described from shell matrix protein analyses of the *Pinctada *genus. The integration of these two methods allowed the global composition of biomineralizing tissue and calcified structures to be examined in tandem for the first time.

**Conclusions:**

This EST study made on the calcifying tissue of *P. margaritifera *is the first description of pyrosequencing on a pearl-producing bivalve species. Our results provide direct evidence that our EST data set covers most of the diversity of the matrix protein of *P. margaritifera *shell, but also that the mantle transcripts encode proteins present in *P. margaritifera *shell, hence demonstrating their implication in shell formation. Combining transcriptomic and proteomic approaches is therefore a powerful way to identify proteins involved in biomineralization. Data generated in this study supply the most comprehensive list of biomineralization-related sequences presently available among protostomian species, and represent a major breakthrough in the field of molluskan biomineralization.

## Background

Mollusk shell is a natural biomaterial made up of a mineral phase - calcium carbonate (CaCO_3_) - and an organic cell-free matrix (proteins, glycoproteins, lipids and polysaccharides) secreted by the external mantle epithelium, the tissue layer underlying the shell. Although this matrix represents less than 2% of the total composition of the shell by dry weight [1], it interacts with the crystal surface to orientate its nucleation and control crystal polymorphism, in the form of aragonite or calcite, in the different structural layers of the shell [2]. The highly organized internal structure of the shell has led to a very interdisciplinary approach to the study of biomineralization. The secretion of shell by mollusks is one of the best examples of a matrix-mediated mineralization process achieved outside living tissues [3,4]. Models of mollusk shell biomineralization have therefore been proposed based on histochemical studies and ultrastructural observations of the shell, combined with biochemical analysis of the extracellular organic matrix. The current "chitin-silk fibroin gel proteins-acidic macromolecules" model proposed by Levi-Kalisman *et al*. [5], updated by Addadi *et al*. [6] and recently reviewed by Furuhashi *et al*. [7], was established from mollusk nacre analysis and involves the major matrix components of the shell. According to this model, the major components of biomineralization are relatively hydrophobic silk proteins and a complex assemblage of hydrophilic proteins (many of which are unusually rich in aspartic acid), highly structured in a polysaccharide β-chitinous framework. These components of the organic matrix are thought to control various aspects of the biomineralization process: the CaCO_3 _crystal polymorphisms (calcite and aragonite) and the microstructures of shell layers [8]. Since the publication of the first complete amino-acid sequence of a nacre-shell protein in 1996 [9], major advances in the field of molecular biology have led to the identification of an increasing number of shell matrix proteins [8]. However, the molecular aspects of shell building are still far from being fully understood.

As marine bivalves are organisms of major economic interest, attention has been turned to the study of their genomics during the last decade [10]. In particular, various sequence-based strategies have been developed for transcriptome studies. Among them, Expressed Sequence Tag (EST) sequencing programs have proven to be an effective method for gene discovery and have been widely used for initiating genomic research in non-model organisms [11]. EST collections provide information on the part of the genome that is expressed, and can be valuable in a number of ways, *e.g*. gene fishing, genome annotation and analysis, discovery of single nucleotide polymorphisms (SNPs), and expression studies such as microarrays. An EST approach to biomineralization offers the opportunity to rapidly identify transcripts encoding secreted shell proteins, proteins specific to the pallial space and proteins implicated in calcium regulation in mantle cells, as well as transcription factors responsible for the regulation of the process. EST programs have recently been developed for aquaculture bivalve species, in particular the Eastern oyster (*Crassostrea virginica*) [12-15], the Pacific oyster (*Crassostrea gigas*) [14,16], and the common blue mussel (*Mytilus galloprovincialis*) [17], but these have mainly been aimed at investigating the mollusk immune response in the context of environmental or genome evolution studies. To date, only five studies report the analysis of EST programs performed on calcifying tissues with the aim of providing more insight into the biomineralization process. Suppression subtractive hybridization (SSH) studies were performed on the bivalve pearl oysters *Pinctada fucata *[18] and *P. margaritifera *[19]. Two other studies, involving the vetigastropod *Haliotis asinina *[20,21] and the bivalve pearl oyster *Pinctada maxima *[21], revealed the high complexity of the calcifying mantle transcriptome, suggesting extensive differences between Bivalvia and Gastropoda in the molecular composition of the organic matrix guiding the deposit of calcium carbonate polymorphs within the shell. The most recent study [22] described the transcriptome of the mantle tissue of *Laturnela elliptica*, focusing on the datamining of genes involved in calcium regulation and shell deposition. Despite these genomic approaches, there is still a small amount of genomic data available on bivalve species and this limits our understanding of the dynamic process of biomineralization.

With the aim of increasing the genomic resources for the pearl-producing bivalve *P. margaritifera*, we conducted a pyrosequencing program to analyze the first EST library produced from the calcifying mantle of this bivalve. Here we report the functional analysis of 276 738 EST sequences, leading to the constitution of a *P. margaritifera *mantle transcript catalog of 82 sequences potentially implicated in the biomineralization process. Further structural characterization of a set of proteins was undertaken in addition to transcript localization and proteomic mass spectrometry analysis of proteins isolated from the shell matrix. Our results show that protein repertoire of the biomineralization process is conserved within pearl oysters, but also provide direct evidence that our EST data set covers most of the diversity of the shell matrix protein in *P. margaritifera *shell.

## Methods

### 1. Mantle RNA Extraction and ESTs library construction

*P. margaritifera *pearl oysters raised in the Vairao lagoon were brought to the Ifremer laboratory in Tahiti, French Polynesia. Total cellular RNA was extracted from 12 mantle samples taken from separate *P. margaritifera *individuals, using TRIZOL^® ^Reagent (Life Technologies) according to manufacturer's recommendations. RNA integrity and purity were assessed in a Bioanalyzer 2100 (Agilent - Bonsai Technologies) and using agarose gel analysis. RNA was quantified using a NanoDrop^® ^ND-1000 spectrophotometer (NanoDrop^® ^Technologies Inc). A pool of 24 μg total RNA (2 μg per sample) was used to construct a cDNA library. Five μg of full-length double-stranded cDNA was processed by the standard Genome Sequencer library-preparation method using the GS DNA Library Preparation Kit to generate single-stranded DNA ready for emulsion PCR (emPCR™). The cDNA library was pyrosequenced using GS FLX technology (454/Roche, http://www.454.com/).

### 2. Contig assembly and functional annotation

EST sequence analysis and assembly were performed by the Skuldtech Company http://www.skuldtech.com. ESTs were assembled into clusters using TGICL (TIGR Gene Indices Clustering tools), freely available on the sourceforge website http://sourceforge.net/projects/tgicl/[23]. Overlapping identity percentage and minimum overlapping length parameters was set to 98% and 60 bp, respectively, in order to obtain highly reliable consensus sequences. Data were archived at NCBI Sequence Read Archive (SRA) under accession SRP002635. ESTs that did not form contigs (singletons) and contigs resulting from the assembly of multiple sequences are referred to as unique sequences. These unique sequences were translated into six reading frames and used as a query to search the non-redundant protein databases available at the National Center for Biotechnology Information (NCBI) using the BlastX algorithm with an E-value ≤10^-3 ^(version # 2.2.15, GenBank release number #166) http://www.ncbi.nlm.nih.gov. Sequences with BlastX hits were manually assigned to the following five sequence categories: known, uncharacterized, predicted, unknown or unnamed, and hypothetical proteins. This classification was based on the information definition lines in each homologous sequence provided by NCBI. All unique sequences with BlastX hits (E-value ≤10^-3^) were functionally annotated using Blast2GO http://www.blast2go.org/[24] by mapping against gene ontology (GO) resources.

### 3. Identification of biomineralization-related proteins in P. margaritifera mantle EST library

Candidate genes from the biomineralization process were locally identified in the *P. margaritifera *mantle ESTs library using BlastX, according to the following parameters: E-value ≤10^-3^, expect feature set to a default value of 10, and low-complexity filter determined by the SEG program [25]. For this purpose, we collected all available sequences regarding biomineralization in mollusks (bivalvia and gastropoda) from the literature or from public databases. The *Pmarg*-Pif nucleotide sequence was obtained by assembling ESTs with an overlapping identity percentage and minimum overlapping length parameters set to 100% and 60 bp, respectively. Motifs and conserved domains of *Pmarg*-Pif protein sequence were used as a query to search the non-redundant protein databases available at the National Center for Biotechnology Information (NCBI) using the BlastP algorithm, according to the following parameters: expect feature set to a default value of 10, and low-complexity filter determined by the SEG program [25]. Sequence alignments were performed using the ClustalW program setting parameters to default for the gap criterions (gap open, no gap end, gap extension, gap distance, pairgap), followed by manual correction with BioEdit software http://www.ebi.ac.uk/Tools/clustalw2/index.html[26]. The presence of signal peptides was inferred using the SignalP 3.0 server http://www.cbs.dtu.dk/services/SignalP/[27]. Conserved domains were identified using Prosite http://www.expasy.ch/prosite/[28]. Percentage identity and biochemical similarity between sequences were calculated using ProtParam http://www.expasy.ch/tools/protparam.html[29]. Repeat detection in protein sequences was performed using RADAR http://www.ebi.ac.uk/Tools/Radar/index.html[30].

### 4. In situ hybridization analyses

#### a) Tissue preparation

*P. margaritifera *mantle tissues were fixed for 24 h in Davidson fixative (22% formalin, 33% ethyl alcohol, 11.5% glacial acetic, 33% sterile sea water), embedded in paraffin wax, and serially sectioned at 7 μm. Sections were collected onto polylysine coated slides (Silane-prep™, Sigma- Aldrich), dried overnight at 60°C and treated with proteinase K (10 μg.mL^-1^) in TE buffer (Tris 50 mM, EDTA 10 mM) at 37°C for 25 min. Slides were then dehydrated by immersion in an ethanol series and air dried. The sections were prehybridized for 1 h at 42°C with 500 μL hybridization buffer (4 × SSC, 50% formamide, 1× Denhardt's solution, 250 μg.mL^-1 ^yeast tRNA, 10% dextran sulfate). The solution was replaced with 120 μL of the same buffer, containing 6 μL of the digoxigenin-labeled sense or antisense probes. The slides were incubated overnight at 42°C for hybridization. The sections were washed twice for 5 min in 2× SSC at room temperature and once for 10 min in 0.4× SSC at 42°C. The detection steps were performed according to manufacturer's instructions (Dig nucleic acid detection kit, Roche Molecular Biomedicals). Slides were finally counter-stained with a solution of Bismark Brown Yellow and mounted in Eukitt. The slides were examined using a DM4000B Leica microscope.

#### b) Specific probe preparation

In order to synthesize probes for *in situ *hybridisation, we used the PeS4 (GACATAGAGAGAGACAGATATGA)/PeAS4 (ATTCACCATTTCCGTTACCGT) primer set, specific to the *pmarg-pearlin *ORF (265bp), and AspF1 (CTCTTACACCAAAATGAAGGGG)/AspR1 (TCCGTCATCATTATCTGC), specific to the *pmarg-aspein *transcript (253 bp). These primers (4 μM final volume) were used in PCR reactions with the iQ™ Supermix (BIO-RAD) and *pmarg-pearlin *full-length cDNA as template. After DNA denaturation at 94°C for 5 min, 35 cycles were run with an MJ-Research thermocycler as follows: 94°C for 30 s; 55°C for 30 s; 72°C for 45 s ended by a final elongation step at 72°C for 10 min. Probes (sense or antisense) were synthesized by asymmetric PCR (using the same amplification program) in the presence of Dig-dUTP (0.7 mM), in a PCR reaction mixture containing a unique primer (sense or antisense, 2 μM final volume), 2 μL of the previously purified PCR fragment (Mini Quick Spin Columns, Roche Diagnostics), a mix of dGTPs-dCTPs-dATPs (200 μM each final), dTTPs (130 μM final), and Taq polymerase (Promega, 2.5 u). Labelling efficiency was assayed using the DIG high prime DNA labelling kit (Roche Diagnostics).

### 5. Purification and identification of proteins from *P. margaritifera *shell

Organic matrix was extracted from fresh shells of *P. margaritifera *specimens aged 3-5 years, after acid acetic decalcification [31]. The acido-insoluble matrix was digested with trypsin prior to reduction and alkylation [32]. Samples were injected into a nano LC-nanoESI-MS/MS system for analysis. Mass spectrometry (MS) was performed using a nanoESI-qQ-TOF, and data acquired automatically using Analyst QS 1.1 software (Applied Biosystems). A 1 s TOF-MS survey scan was acquired over 400-1600 amu, followed by three 3 s product ion scans over a mass range of 65-2000 amu. The three most intense peptides, with a charge state of two to four above a 30 count threshold, were selected for fragmentation and dynamically excluded for 60 s with ± 50 mmu mass tolerance. The collision energy was set by the software according to the charge and mass of the precursor ion. The MS and MS/MS data were recalibrated using internal reference ions from a trypsin autolysis peptide at *m/z *842.51 [M + H]^+ ^and *m/z *421.76 [M + 2H]^2+^. Protein identification was done using the Mascot database-searching software (Matrix Science, London, UK; version 2.2.04) using our database of the pyrosequencing-based EST mantle library from *P. margaritifera*. Carbamidomethylation and oxidation were set as fixed and variable modifications, respectively. The mass tolerance was set to 0.5 Da and the MS/MS tolerance to 0.2 Da.

## Results and Discussion

During recent decades, high-throughput techniques have been used to examine a broad range of physiological processes and applications in diverse fields of biology [33,34]. To examine the biomineralization process in pearl oyster *P. margaritifera*, we performed transcriptome pyrosequencing of its calcifying tissue combined with a proteome analysis of the shell.

### 1. Transcriptome analysis of *P. margaritifera* calcifying mantle

#### a) Generation of ESTs and contig assembly

We constructed and pyrosequenced a *P. margaritifera *mantle cDNA library, resulting in the production of 276 738 sequences of an average size of 234 bp (Table 1). Sequences in the library ranged from 33 to 406 bp, with the most abundant group of sequences (70%) in the 225-290 bp range and only 3% of the sequences longer than 300 bp. The 276 738 ESTs were assembled into clusters using TGICL, which gave 19 257 contigs and 57 533 singletons. Our *P. margaritifera *mantle EST collection thus contains 76 790 unique sequences (Table 1). The number of EST sequences generated here using pyrosequencing is similar to numbers obtained in other transcriptome pyrosequencing based studies [35]. The May 2010 GenBank release only contained 116 sequences from *P. margaritifera *including both the "nucleotide" and "EST" sections, and a total of 15 742 sequences from the genus *Pinctada *as a whole. Once released on public databases, the present 76 790 mantle unique sequences will account for 99.8% of all sequences available for *P. margaritifera *and 83% of all sequences available for the genus *Pinctada*. Pyrosequencing is, therefore, both a rapid and powerful way to dramatically increase transcriptomic resources for non-model organisms lacking detailed genomic data.

**Table 1:** Summary statistics for pyrosequencing and annotation of *P. margaritifera *mantle ESTs

Feature	Number	Percentage
Total number of ESTs sequenced	276 738	-
Average lenght of ESTs (bp)	234	-
Number of assembled EST	219 205	79.2%
Number of contigs	19 257	-
Number of singletons	57 533	20.8%
Number of unique sequences	**76 790**	-
Ratio of singletons per unique sequences	-	74.9%

Number of contigs containing 2 ESTs	8 717	45.3%
Number of contigs containing 3 ESTs	3 419	17.8%
Number of contigs containing 4 ESTs	1 779	9.2%
Number of contigs containing 5 ESTs	1 119	5.8%
Number of contigs containing > 6 ESTs	4 223	21.9%

Number of annotated unique sequences:	**29 479**	**38.4%**
- Known protein	13 064	44.3%
- Uncharacterized	6 010	20.4%
- Predicted	4 795	16.3%
- Unknown, Unnamed	2 880	9.8%
- Hypothetical protein	2 730	9.3%
Number of annotated contigs	10 007	52.2%
Number of annotated singletons	19 472	33.8%

Of the 19 257 contigs, 8717 (45.3%) contained 2 ESTs, 3419 (17.8%) contained 3 ESTs, 1779 (9.2%) contained 4 ESTs, 1119 (5.8%) contained 5 ESTs, and 4223 (21.9%) contained more than 6 ESTs (Table 1). In our study, 79.2% of the 276 738 ESTs were successfully assembled and remaining singletons only represented 20.8% of the reads, and a large part (74.9%) of the 76 790 unique sequences was singletons. In other recent 454 transcriptome studies, results showed that the remaining singletons represented 10 to 40% of the reads [36,37]. It has already been observed that many ESTs resulting from deep sequencing of transcriptomes with 454 sequencing technology fail to assemble [38]. These unassembled singletons could result from sequencing errors, contaminants from other sources, or can even from technical difficulties in assembling with overlaps that are too short in length or which contain highly repeated sequences. Interestingly, however, these singletons can also represent rare transcripts of genes expressed at low levels [39], and therefore constitute an interesting source of genomic data.

#### b) Putative identities of ESTs

BlastX searches of the 76 790 unique sequences in the non-redundant protein databases available at the National Center for Biotechnology Information (NCBI) revealed 29 479 (38.4%) significant matches (E-value ≤10^-3^). Among these 29 479 matches, 13 064 (44.3%) are known proteins, but 6010 are uncharacterized (20.4%), 4795 are predicted (16.3%), 2880 are either unknown or unnamed (9.8%), and 2730 are hypothetical proteins (9.3%) (Table 1). This apparently low rate of identification is common among mollusk EST databases, with which rates usually range from 15 to 40% [15,17,22,40], although this is lower than for vertebrates [41], or even EST collections from model plants [42].

Although the lack of annotation can result from the difficulty of annotating some short length sequences, it can largely be explained by the lack of sequences available for mollusk species, and by the fact that a vast majority of genes on public databases come from taxa (in particular vertebrates species) whose amino acid sequences show great divergence with those of protostomians.

#### c) Functional Gene Ontology annotation

**Figure 1:**
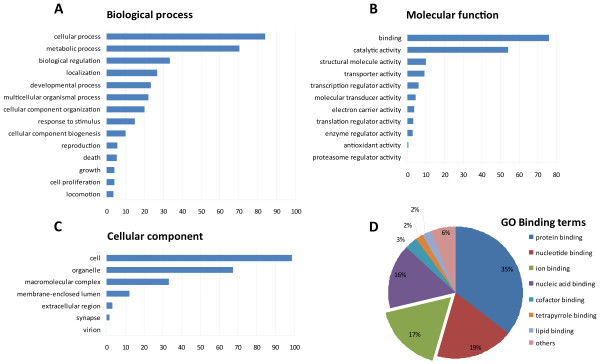
**Gene Ontology annotation of annotated unique sequences (singletons and contigs) from *P. margaritifera***. Distribution (%) of gene ontology terms among the annotated unique sequences. GO ontologies are represented as general function categories. Among the 10 004 unique sequences with GO assignation, 5976 (59.7%) are involved in biological processes (A), 6855 (68.5%) have molecular functions (B) and 5737 (57.3%) are cellular components (C). Specialized terms of the binding category repartition (D) shows 17% of sequences implicated in ion binding.

Gene Ontology (GO) assignment was carried out on unique sequences in order to categorize transcripts from *P. margaritifera *mantle by putative function. The GO project provides a structured and controlled vocabulary of terms (ontologies) for describing gene product characteristics and gene product annotation data [43]. In our study, 10 004 unique sequences (13.0%) were successfully assigned to one or more GO terms. Among these, following the functional classification with the three unrelated GO ontologies, 5976 (59.7%) are involved in biological processes, 6855 (68.5%) have molecular functions and 5737 (57.3%) are cellular components. For each of these three ontologies, annotated sequences are mainly distributed among two or three of the general term categories. Within the 5976 unique sequences involved in biological processes, 5006 (83.8%) and 4191 (70.1%) are dedicated to cellular processes and metabolic processes, respectively (Figure 1A). Similarly, in the molecular functions sub-ontology, 5208 (76.0%) and 3704 (54.0%) of the 6855 unique sequences have binding and catalytic activity, respectively (Figure 1B). Finally, of the 5737 unique sequences predicted to be cellular components, 5656 (98.6%) and 3868 (67.4%) are related to cell and organelle components, respectively (Figure 1C). These results constitute common features among EST databases available from marine organisms, and in particular mollusks [40,44].

The distribution of the sequences between specialized terms in the binding section of the molecular function category showed that the greatest numbers fell under protein-binding (35%) and nucleotide-binding (19%). Interestingly, the third greatest number of the binding section fell into ion-binding (17%) (Figure 1D). Biomineral crystal matrix macromolecules play a key role in biologically-controlled biomineralization processes. *In vitro *crystallization experiments, microscopic and analytical methods revealed stereochemical properties of matrix proteins, which allow them to bind calcium ions and calcium carbonate, and therefore perform framework building and crystal growth during the construction of the molluskan shell[45-48]. A significant proportion of sequences in our mantle EST collection are implicated in binding, and particularly in ion binding. This result is consistent with observations from a previous study performed on the calcifying mantle of the bivalve *L. elliptica *[22]. We therefore hypothesize that this classification could be a pattern typical of tissues of a secretory nature implicated in biomineralization processes.

### 2. Identification of transcripts encoding proteins involved in the biomineralization process of *P. margaritifera*

#### a) Identification of a catalogue of 82 proteins potentially involved in the biomineralization process

To obtain an integrated view of the transcriptional events of the biomineralization process in *P. margaritifera *mantle, we made BlastX searches with our EST mantle library focusing on proteins known to be involved in these mechanisms. For this purpose, we first collected all available sequences regarding biomineralization in calcifying invertebrates from the literature or from public databases. In mollusks, we found 140 bivalve and 103 gastropod proteins potentially implicated in biomineralization processes. These 243 molluskan sequences were isolated from shell or mantle tissue in previous studies, using either biochemical or molecular biology approaches. BlastX searches of the 140 bivalves and 103 gastropods proteins in our EST database revealed 121 and 56 significant matches (E-value ≤10^-3^), respectively. Analyzing these 177 sequences together with sequences from our EST library, we identified 82 *P. margaritifera *non-redundant unique sequences potentially implicated in the biomineralization process. Among these, 69 and 13 sequences could be recovered by homology with sequences from bivalve and gastropod, respectively.

Among the 69 unique *P. margaritifera *transcripts that were recovered by homology with the bivalve sequences, 55 sequences were obtained by homology with sequences from the *Pinctada *genus (Additional file 1). The overall identity percentage between *P. margaritifera *protein sequences potentially implicated in the biomineralization process and protein sequences from the *Pinctada *genus is ranging from 24% (C-type lectin 2 from *P. fucata*) to 95% (Ferritin-like protein from *P. fucata*). This level of identity is similar to percentages already observed for homolog proteins from the N66/Nacrein and N14/N16 families [49-51]. The N66 sequence from *P. maxima *and Nacrein sequence from *P. fucata *(*P. maxima *N44 homolog sequence) displayed identity percentages of 82% and 69%, respectively, with *P. margaritifera *homolog sequence. Similarly, the N14 sequence from *P. maxima *and N16 sequence from *P. fucata *displayed an identity percentage of 93% and 71% respectively with *P. margaritifera *homolog sequence, Perline matrix protein. Considering all sequences from the *Pinctada *genus, the identity percentage seems to be higher between *P. margaritifera *and *P. maxima *sequences than between *P. margaritifera *and *P. fucata *sequences.

Extending our analysis to biomineralization proteins from other bivalves led us to the identification of the 14 remaining sequences out of the 69 unique *P. margaritifera *transcripts that were recovered by homology with the bivalve sequences (Additional file 1). The overall identity percentage between *P. margaritifera *protein sequences potentially implicated in the biomineralization process and protein sequences from the other bivalves ranges from 28% (EP protein precursor from *Mytilus edulis*) to 58% (bone morphogenic protein type 2 receptor from *Crassostrea gigas*). This level of identity is lower than that observed between proteins within the *Pinctada *genus, except for proteins implicated in calcium regulation or signal transduction. For example, Calmodulin sequences from *Hyriopsis schlegelii *(genbank accession number: ACI22622) displayed an identity percentage of 99% with the *P. margaritifera *homolog sequence.

Finally, we identified 13 *P. margaritifera *unique sequences by homology with sequences of gastropod (Additional file 1). The overall identity percentage between *P. margaritifera *protein sequences potentially implicated in the biomineralization process and protein sequences from gastropods ranged from 27% (Veliger mantle 1 from *H. asinina*) to 100% (Calmodulin from *Conus cuneolus)*. Interestingly, some sequences homologous to abalone (*H. laevigata*) proteins could be found in our EST database, namely Perlucin [52,53], Perlustrin [53,54] and Perlawpin [55] from *Haliotis laevigata*. Perlucin, Perlustrin and Perlwapin sequences were obtained by direct protein sequencing of proteins purified from the nacreous layer of abalone shell. All of the *P. margaritifera *homolog sequences for each of these 3 proteins found in the *P. margaritifera *EST library display the same motif and numerous conserved cystein positions as in the sequences from *H. laevigata*. Perlucin is a 155-amino acid protein which exhibits similarities with calcium dependent lectins (C-type). The *P. margaritifera *homolog sequence for Perlucin (*Pmarg-*perlucin) is not a complete sequence. However, of the 6 cysteins present in the abalone sequence, 3 are conserved between *Pmarg-*perlucin and Perlucin sequences. Moreover, *Pmarg-*perlucin displays an E-value of 9.00E-9 and an identity percentage of 38% (27/71 a.a.) with Perlucin and also has a C-type lectin domain. Perlustrin is a small protein (84 a.a.) with similarities to vertebrate insulin-like growth factor-binding protein (IGF-BP) sequences. The *P. margaritifera *homolog sequence for Perlustrin (*Pmarg*-perlustrin) is a complete 142-amino acid sequence with an E-value of 7.00E-6, and 39% (25/64 a.a.) identity with Perlustrin; it also exhibits a insulin-like growth factor binding proteins (IGFBPs). On the 12 cysteins scattered across the Perlustrin sequence, 11 (of the 14 cysteins of *Pmarg*-perlustrin) are conserved between *Pmarg*-perlustrin and the Perlustrin sequences. Finally, the Perlwapin protein consists of 134 amino acids that contain 3 repeats of 40 amino acids very similar to the well-known whey acidic protein (WAP) domains. The *P. margaritifera *homolog sequence for Perlwapin (*Pmarg*-perlwapin) is a complete 139-amino acid (a.a.) sequence with an E-value of 2.00E-11, 37% identity (40/107 a.a.) with Perlwapin, and two WAP domains. Out of the 25 cysteins spread along the Perlwapin sequence, all 14 cysteins of *Pmarg*-perlwapin are conserved between the *Pmarg*-perlwapin and Perlwapin sequences. These results would suggest that Perlucin, Perlustrin and Perlwapin are present in *P. margaritifera*. Previous studies have shown that there are significant differences in the molecular mechanisms in different mineralizing species and, therefore, between the proteins they use. Such differences may even exist among species that are phylogenetically very close, like the Mollusca. The cause of this "evolvability" remains a controversy, and it is still uncertain whether the biomineralization "molecular tool box" required for shell construction is inherited from an ancestral function, or whether this ability is the result of an adaptive convergence. Recent studies have explicitly demonstrated that shell or skeletal proteins had evolved independently among metazoans [8,21,56]. However, the identification of homolog proteins between bivalvia and gastropoda could support the idea that at least some of the shell component could have appeared early in the evolution of the molluscan phylum.

Taken together, this candidate approach allowed us to isolate 82 unique sequences potentially implicated in the biomineralization process in *P. margaritifera*. This study considerably increases the amount of transcriptomic data available in this field, making *P. margaritifera *the best documented marine protostomian with regard to biomineralization.

#### b) Identification of proteins from the "chitin-silk fibroin gel-acidic macromolecule" model

Mollusk shell construction is the result of biologically-controlled mineralization, a highly dynamic process mediated by an extracellular organic matrix secreted by the mantle epithelium [3]. Histochemical studies and ultrastructural observations of the shell, together with biochemical analysis of the extracellular organic matrix, provided a better understanding of shell structure and led to the identification of proteins composing it, thereby allowing mollusk shell biomineralization models to be developed. The currently accepted "chitin-silk fibroin gel-acidic macromolecule" model involves the major matrix components of the shell, *i.e*. relatively hydrophobic silk proteins plus a complex assemblage of hydrophilic proteins (many of which are unusually rich in aspartic acid), highly structured in a polysaccharide β-chitinous framework [6].

In our study, beyond the consideration of protein homologies between species, it is interesting to note that our *P. margaritifera *EST mantle library includes sequences coding for proteinaceous components of the matrix following this model. Firstly, a sequence showing 78% identity with MSI60 from the silk fibroin matrix component could be retrieved. MSI60 is an insoluble framework protein purified from the nacreous layer of the shell [57] and expressed in the more dorsal region of the mantle [58]. Poly-Ala and poly-Gly blocks conferring MSI60 homologies with spider silk fibroins are present in the *P. margaritifera *homologous sequence. MSI31 [57] and Shematrins [59], displaying silk/fibroin-like domains, could be also retrieved. Secondly, a sequence showing 87% identity with the unusually acidic protein Aspein from *P. fucata *could be recovered in the EST database from *P. margaritifera *[60]. This sequence homologous to Aspein is the first extremely acidic shell protein identified in *P. margaritifera*. In *P. fucata*, Aspein is specifically expressed in the mantle region, which secretes the calcite prism matrix [58]. The main body of this protein includes a high proportion of Asp (60.4%) punctuated with Ser-Gly dipeptides, which are conserved in the *P. margaritifera *homologous sequence. Finally, recent electron microscopy studies on nacre have detected the presence of chitin in the shell of *P. margaritifera *[61], and chitin synthase gene has been cloned from *P. fucata *[62], *Atrina rigida *and *Mytilus galloprovincialis *[63]. A *P. margaritifera *homolog sequence of chitin synthase from this species could be retrieved, revealing that chitin synthase sequences are well conserved among bivalves. More precisely, the chitin synthase sequences from *Atrina rigida *and *Mytillus galloprovincialis *displayed identity percentages of 91% and 84%, respectively, with the homologous *P. margaritifera *sequence.

Taken together, searches realized on the EST mantle library allowed us to identify proteinaceous components of the calcifying matrix from *P. margaritifera*. These results demonstrate how EST-based studies are a powerful way of dramatically increasing knowledge about proteins implicated in the biomineralization process, which constitutes an important prerequisite for establishing relevant biomineralization models.

#### c) Pmarg-Pif encodes an homolog of Pif-177 from P. fucata, a protein involved in nacre formation

**Figure 2:**
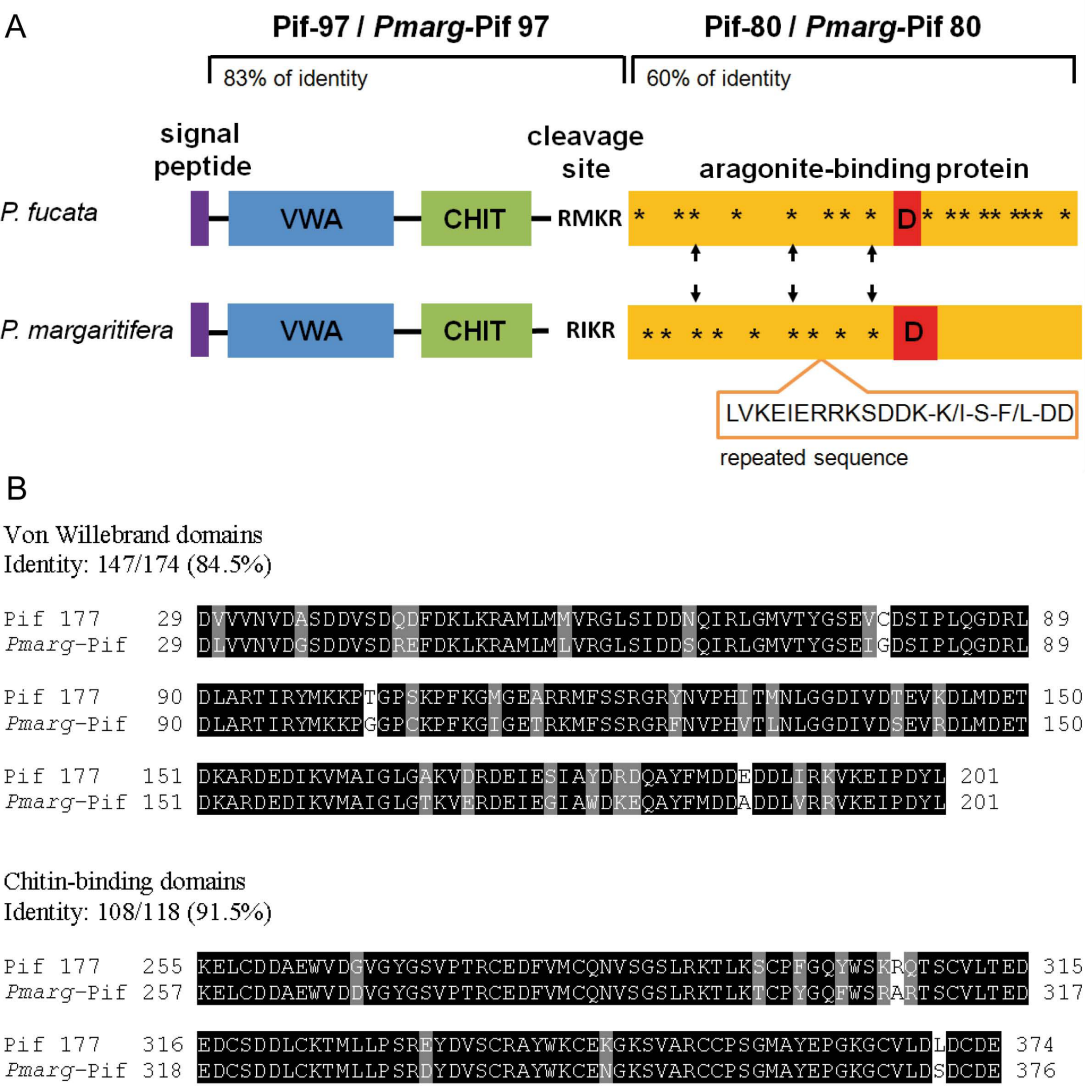
**Pif-177 and *Pmarg*-Pif protein sequence comparison**. (A). Schematic representation of Pif-177 and *Pmarg-*Pif protein structures. Pif-97 consists of 525 amino acids (from a.a. 23 to 547) and *Pmarg-*Pif-97 consists of 536 amino acids (from a.a. 23 to 558). Pif-80 consists of 460 amino acids (from a.a. 548 to 1007) and *Pmarg-*Pif-80 consists of 456 amino acids (from a.a. 559 to 1014). Purple box is the signal peptide. Blue VWA box is von-Willebrand type A domain. Green CHIT box is a chitin-binding domain. Yellow box is aragonite-binding protein. The four-amino-acid motifs (DD-R/K-R/K) are indicated by asterisks, and arrows indicate motifs at conserved positions between the two sequences. Red box, in the aragonite-binding protein, indicates the cluster of acidic amino acid residues. (LVKEIERRKSDDK-K/I-S-F/L-DD) consensus motif repeated three times in *Pmarg-*Pif is boxed under aragonite-binding protein. (B) Alignments of the Pif-177 and *Pmarg-*Pif amino acid sequences of the von Willebrand type A domain and chitin-binding domain. Residues shaded in black are identical residues, and grey positions indicate conserved residues. Sequences are preceded and followed by numbers indicating the domain position in the entire sequence. Percentage of identity are indicated.

Pif-177 is an acidic matrix protein that was identified in *P. fucata *nacre shell and is known to specifically bind to aragonite crystals. Results from immunolocalization, RNA interference and *in vitro *calcium carbonate crystallization strongly indicate that Pif-177 regulates nacre formation; making Pif-177 the first mineralization protein in this species whose function was identified *in vivo *[64]. A Pif-177 homolog, which we named *Pmarg*-Pif, was identified in the *P. margaritifera *mantle EST library using BlastX. A comparison between *Pmarg*-Pif and Pif-177 protein structures is shown in figure 2A. A consensus furin cleavage site at position 555-558 (RIKR) was identified in the *Pmarg*-Pif sequence. In *P. fucata*, a similar furin-cleavage domain, observed between amino acid positions 544-547 (RMKR) is required for Pif-177 cleavage into Pif-80 and Pif-97. This suggests that, as with Pif-177, *Pmarg*-Pif cDNA is very likely to encode a precursor protein that will be post-translationally cleaved to produce Pif-97 and Pif-80 homologs (which we named *Pmarg*-Pif-97 and *Pmarg*-Pif-80, respectively). Alignments of *P. fucata *and *P. margaritifera *Pif sequences revealed 83.0% of identity between Pif-97 and *Pmarg*-Pif-97 domains, and 60.0% identity between Pif-80 and *Pmarg*-Pif-80. Like Pif-97 and Pif-80, *Pmarg*-Pif-97 and *Pmarg*-Pif-80 are acidic proteins. *Pmarg*-Pif-97 consists of 536 amino acid residues, including a high proportion of charged amino acid residues, Asp (15.3%), Glu (7.1%), Lys (10.3%), and Arg (6.0%), with a calculated isoelectric point (p*I*) value of 4.7. This sequence contains two conserved domains: a von Willebrand type A (VWA) domain and a chitin-binding domain, similar to those in Pif-97 (figure 2B). There are also 22 Cys residues, of which 21 are conserved between the two homologs. *Pmarg*-Pif-80 consists of 456 amino acid residues, and also contains a high proportion of charged amino acid residues, Asp (26.1%), Glu (5.0%), Lys (16.2%), and Arg (11.6%), with a calculated isoelectric point (p*I*) value of 5.13. *Pmarg-*Pif-80, like Pif-80, displays a high Asp ratio and may, therefore, be involved in aragonite-binding processes, since Pif-80 was shown to bind aragonite crystals and Pif-177 to be implicated in the regulation of nacre formation. A cluster of acidic amino acid residues is also present near the center of the molecule, but this shows a higher number of Asp residues (14) in *Pmarg*-Pif-80 than in Pif-80 (10). Only 9 repeats of the four-amino-acid motif (DD-R/K-R/K) could be found before the cluster of acidic amino acid residues in *Pmarg-*Pif-80, whereas 17 are found scattered throughout Pif-80 sequence, and only three of these are conserved in the same position between the two sequences. Interestingly, an 18 amino acid residue sequence (LVKEIERRKSDDK-K/I-S-F/L-DD) is repeated three times (742-816) in the *Pmarg-*Pif-80 sequence. This highly charged amino acid sequence could not be retrieved in the Pif-80 sequence, and BlastP results showed no homology with any other protein in the public database. Considering that this consensus sequence is localized in a protein sequence suspected to play a role in aragonite binding in *P. fucata*, it potentially constitutes an interesting new motif with regard to biomineralization processes.

Taken together, the numerous conserved sequence motifs, conserved cystein residue positions, charged amino acid residue composition and common isoelectric properties between *Pmarg-*Pif-97 and Pif-97 support the hypothesis that *Pmarg*-Pif might have a similar activity to Pif-177, and regulate nacre formation in *P. margaritifera*. However, the presence of the repeated 18 amino acid residues sequence specific to *Pmarg-*Pif-80 and the distinct number of repeats of the four-amino-acid motif (DD-R/K-R/K) between *Pmarg-*Pif-80 and Pif-80 also suggest that *Pmarg-*Pif-80 might have a function specific to *P. margaritifera*. Considering these features, further research needs to be undertaken in order to investigate *Pmarg-*Pif function and its role in the biomineralization process.

### 3. Expression pattern of biomineralization-related protein transcripts

**Figure 3:**
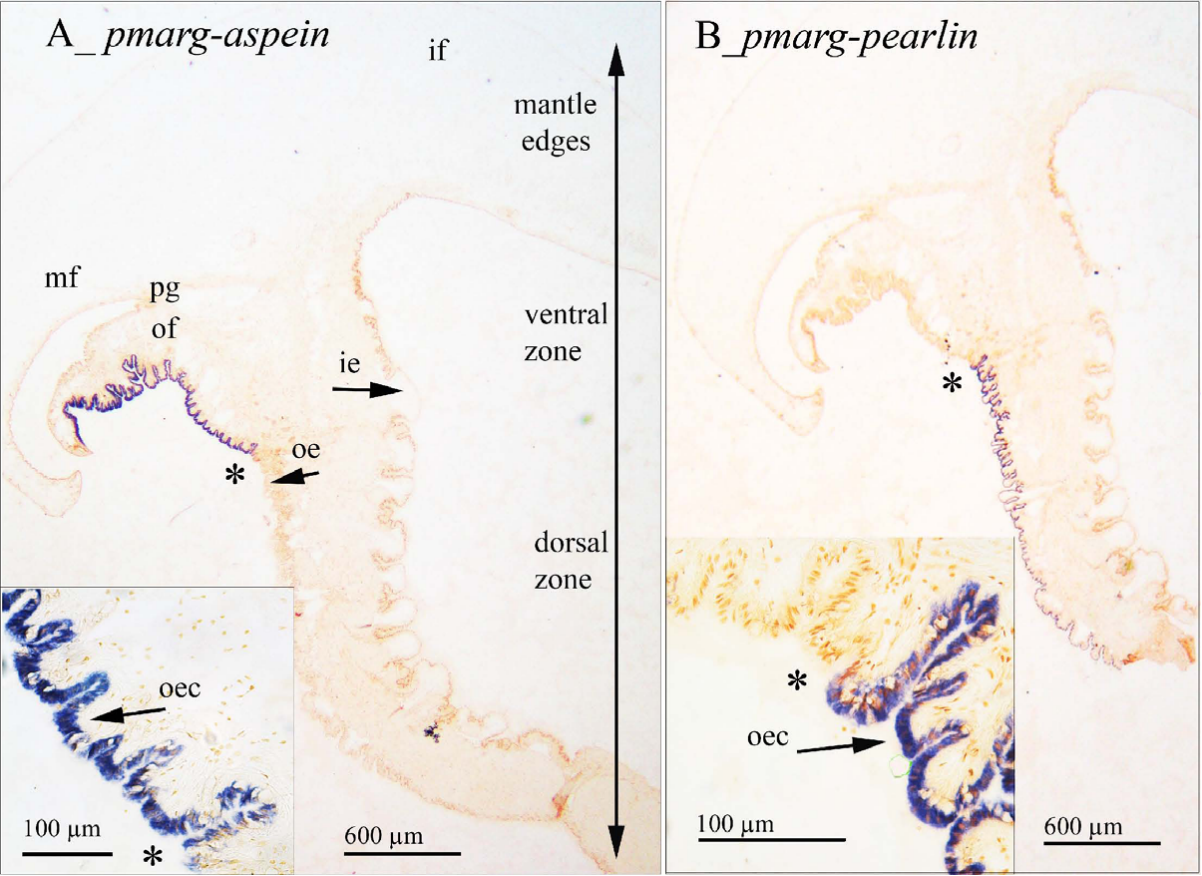
**Localization of pmarg-*pearlin *and *pmarg-aspein *gene transcripts in *P. margaritifera *mantle tissue by *in situ *hybridization**. Paraffin-embedded sections of oyster tissues were hybridized with antisense or sense single stranded cDNA probes labeled with digoxigenin and revealed using alkaline phosphatase-conjugated antibodies. Positive cells are stained in dark blue, sense probes showed no hybridization (data not shown). Stained cells enlargements are shown in A and B insets where scale bars are indicated. The expression partition limit is symbolized by a *. if: inner fold; mf: middle fold; of: outer fold; pg: periostracal groove; oe: outer epithelium; ie: inner epithelium; oec: outer epithelial cell.

In order to focus on biomineralization processes, transcript localization was performed using isolated transcripts from the *P. margaritifera *EST library. We selected two highly documented proteins implicated in biomineralization in the *Pinctada *genus: the Aspein and N14/N16/Pearlin families. In adult oysters, the tissue responsible for shell mineralization is the mantle outer epithelium. This mantle can be divided into several regions from the more proximal (dorsal) zone to the more distal (ventral to mantle edge) zone. Studies have shown that this zonation can be associated with distinct gene expression patterns, suggesting a functional partition following the dorso-ventral axis, which might be involved in production of specific calcium carbonate polymorphs [57,65]. Our *in situ *analysis revealed that these transcripts were specifically localized in the outer epithelium of the mantle known to be bearing mineralizing cells (figure 3). More interestingly, these transcripts were localized in two distinct areas of the outer epithelium, the dorsal zone for *pmarg-pearlin *and the ventral zone for *pmarg-aspein. Aspein *and *pearlin *genes are known to produce proteins specific to the nacre and prismatic layers of the shell, respectively [49,58,60,66]. Our observations confirm the functional subdivision within the pearl oyster mantle outer epithelium, *pmarg-pearlin *transcripts being specific to aragonitic nacre-forming cells and *pmarg-aspein *transcripts being specific to calcitic prism-forming cells.

### 4. Mantle transcripts encode proteins identified in *P. margaritifera *shell

Using the *P. margaritifera *EST mantle library, identification of shell matrix proteins was attempted by a complementary proteomic approach. The shell matrix proteins, extracted from decalcified shell powder, were digested with trypsin and the resulting peptides were analysed by MS/MS mode mass spectrometry. The raw MS/MS data were directly interrogated against the EST data set using Mascot software. After careful observation of the MS/MS data on the 50 first most intensive peptides, we estimated that almost all the main peptides analysed led to contig identification. We only considered matching proteins that presented at least 2 unambiguously identified peptides, *i.e*. those presenting individual scores superior to the threshold (calculated value of 32).

This shotgun proteomic approach allowed us to identify more than 30 contigs, but only 13 proteins presented homologies to previously characterized mollusk shell proteins (Table 2). This list of proteins contains almost all of the shell proteins previously described before from shell matrix protein analysis of the *Pinctada *genus. [8]. Among them, PFMG10, Linkine, MPN88 and KRMPs were only characterized at the transcriptional level until now, and direct evidence of their implication in shell biomineralization process was lacking. Our data shows that they are definitely part of the calcifying matrix, which is embedded within the biomineral structures in the shell of *P. margaritifera*. We noticed that the few missing matches from the list of the biomineralization-related protein of the *Pinctada *genus. [8] - Aspein, MSI7 and Prisilkin-39 - exhibit sequences that are remarkably deficient in trypsin cleavage sites, making them unsuitable for standard proteomic detection. However, it is worth noting that homologs of the latter proteins are observed in the EST mantle library (Additional file 1).

**Table 2:** Protein identification in the shell matrix of *P. margaritifera *by a proteomic approach.

Protein	Matching peptides	Mascot score	Homolog protein	Accession no.	Identity	E-Value	Species
***Pmarg*-Pif177**	26	1402	Pif177*	C7G0B5	72%	0.0	*P. fucata*
***Pmarg*-N66**	16	759	N66* Nacrein*	Q9NL38 Q27908	81% 67%	0.0 1 e-123	*P. maxima* *P. fucata*

***Pmarg*-PFMG10**	9	519	PFMG10	Q45TK0	56%	4 e-52	*P. fucata*

***Pmarg*-Linkine**	6	278	Linkine	B5KFE5	100%	4 e-62	*P. margaritifera*

***Pmarg*-Pearlin**	6	183	Perline* N14/Pearlin*	Q14WA6 Q9NL39	96% 92%	2 e-85 2 e-82	*P. margaritifera* *P. maxima*

***Pmarg*-Shematrin-2**	4	340	Shematrin-8 Shematrin-2*	B5KFD0 Q1MW95	96% 68%	1 e-142 2 e-85	*P. margaritifera* *P. fucata*

***Pmarg*-Pfty-1**	4	285	Pfty-1*	A1IHF0	59%	1 e-146	*P. fucata*

***Pmarg*-Shematrin-5**	4	248	Shematrin-5	Q1MW92	54%	1 e-68	*P. fucata*

***Pmarg*-MSI60**	3	181	MSI60*	O02402	78%	1 e-120	*P. fucata*

***Pmarg*-MPN88**	3	126	MPN88	B7X6S0	47%	2 e-87	*P. fucata*

***Pmarg*-KRMP-2**	3	121	KRMP-11 KRMP-2	A7X103 C4TPC8	88% 56%	1 e-30 7 e-14	*P. margaritifera* *P. fucata*

***Pmarg*-Shematrin-1**	2	189	Shematrin-9 Shematrin-1*	B5KFD1 Q1MW96	98% 65%	4 e-99 8 e-62	*P. margaritifera* *P. fucata*

***Pmarg*-Prismalin-14**	2	149	Prismalin-14*	Q6F4C6	68%	5 e-40	*P. fucata*

The trypsin-digest peptides were separated on nano-LC, prior to nanoESI-qQ-TOF analysis. The MS/MS spectra were used for searching against the pyrosequencing based EST mantle library with Mascot software. We only considered proteins that presented at least 2 matching peptides. Mascot protein scores are indicated together with the number of unique peptides that matched to the sequence. The homologies were determined by BlastP interrogations against UniProtKB/Swiss-Prot protein database (January 2010) using the UniProt on-line tool http://www.uniprot.org, setting parameters to default. We only show here the best matches for the contigs which protein sequences are the homologues of already known mollusk shell proteins. We notice that all matches are from *Pinctada *genus origin.

* indicates proteins which the occurrence in shell matrix was previously demonstrated by direct biochemical characterization.

Our proteomic analysis enabled us to retrieve *in silico *all the sequences from *P. margaritifera *involved in the biomineralization process already published on databases in our peptide library, and we were also able to find a match in our database for all proteins experimentally found from *P. margaritifera *shell in our EST library. These results demonstrate that our EST data set covers most of the diversity of the matrix protein of the *P. margaritifera *shell.

## Conclusion

This global approach combining transcriptome and proteome analysis of *P. margaritifera *calcifying mantle and shell is the first description of a pyrosequencing program performed on a pearl-producing bivalve species. It led to the functional analysis of 276 738 EST sequences, with the constitution of a *P. margaritifera *mantle transcripts catalog of 82 sequences potentially implicated in the biomineralization process. Our results showed that the biomineralization protein repertoire is conserved within pearl oysters, but also provided direct evidence that our EST data set covered most of the diversity of *P. margaritifera *shell matrix protein. These observations clearly demonstrate the high efficiency of this pyrosequencing-based EST library in accurately identifying shell proteins, in combination with shotgun proteomic analysis and automated database searches. These data represent the most comprehensive list of biomineralization-related sequences available among protostomian species, and represent a major breakthrough in the field of molluskan biomineralization.

## Authors' contributions

NCL and CM wrote the grant proposal. NCL, CM and YG conceived the project. CJ, BM and DP contributed to conception and design of the experiments. DP coordinated the construction, sequencing and analyses of the EST library. CJ performed RNA extraction and EST library construction, assisted with the functional annotations, analysis and interpretation of data and drafted the manuscript. BM and IZC carried out proteomic analysis of the shell matrix proteins. LM and FP performed contig assembly and functional annotation. CM performed *in situ *hybridization analyses. YG, CM and DP contributed to supervision of the work and critical review of the manuscript. All authors read and approved the final manuscript.

## Supplementary Material

Additional file 1 **Table S1**: Summary of BlastX results of biomineralization-related protein in the EST *P. margaritifera *mantle database. A catalogue of 82 *P. margaritifera *mantle transcripts potentially implicated in the biomineralization process was constructed using BlastX (E-value < 10^-3^) with selected protein sequences identified from mollusks (bivalvia and gastropoda). Click here for file
