# Supplementary material for: Transcriptome and proteome analysis of Pinctada margaritifera calcifying mantle and shell: focus on biomineralization
Source: BMC Genomics. 2010 Nov 1;11:613. doi: 10.1186/1471-2164-11-613 (PMC3091754; doi:10.1186/1471-2164-11-613)
Supplement: Additional file 1 — Table S1: Summary of BlastX results of biomineralization-related protein in the EST P. margaritifera mantle database. A catalogue of 82 P. margaritifera mantle transcripts potentially implicated in the biomineralization process was constructed using BlastX (E-value < 10-3) with selected protein sequences identified from mollusks (bivalvia and gastropoda). [file 1471-2164-11-613-S1.DOC]

| ***P. margaritifera* sequence (Subject)** | **Protein Name (Query)** | **Species** | **Genus** | **GenBank Protein Accession Number** | **Identities (Query /Subject)** | **Homolgy Percentage** | **BlastX E-value** | **Protein Length (Query)** |
| --- | --- | --- | --- | --- | --- | --- | --- | --- |
| Contig_90c_305_4 | pfGbeta1 | *Pinctada fucata* | Bivalvia | Q5GIS3 | 340/341 | 99 | 0.0 | 341 |
| Contig_90c_615_2 | Chitin synthase / PfCHS1 | *Pinctada fucata* | Bivalvia | BAF73720 | 978/1007 | 97 | 0.0 | 2276 |
| Contig_90c_501_4 | calreticulin | Pinctada fucata | | Bivalvia | | --- | | ABR68546 | 335/371 | 90 | 0.0 | 414 |
| Contig_90c_221_4 | N66 matrix protein | *Pinctada maxima* | Bivalvia | BAA90540 | 504/611 | 82 | 0.0 | 568 |
| Contig_90c_71_3 | Pif 177 | *Pinctada fucata* | Bivalvia | BAH97338 | 718/967 | 74 | 0.0 | 1007 |
| Contig_90c_2232_6 | N33 | *Pinctada maxima* | Bivalvia | [ACS50182](http://www.ncbi.nlm.nih.gov/protein/240265528?ordinalpos=1&itool=EntrezSystem2.PEntrez.Sequence.Sequence_ResultsPanel.Sequence_RVDocSum) | 298/374 | 79 | 1,00E-175 | 329 |
| Contig_90c_665_4 | 67kD laminin receptor precursor | *Pinctada fucata* | Bivalvia | [ABO10190](http://www.ncbi.nlm.nih.gov/protein/126362082?ordinalpos=1&itool=EntrezSystem2.PEntrez.Sequence.Sequence_ResultsPanel.Sequence_RVDocSum) | 289/299 | 96 | 1,00E-169 | 301 |
| Contig_90c_942_2 | Shematrin-9 | *Pinctada margaritifera* | Bivalvia | ABO92761 | 255/276 | 92 | 1,00E-152 | 332 |
| Contig_90c_131_4 | Tyrosinase-like protein 1 (Pfty1) | *Pinctada fucata* | Bivalvia | BAF42771 | 281/470 | 59 | 1,00E-152 | 533 |
| Contig_90c_148_5 | Shematrin-8 | *Pinctada margaritifera* | Bivalvia | ABO92760 | 244/252 | 96 | 1,00E-144 | 416 |
| Contig_90c_187_3 | N44 | *Pinctada maxima* | Bivalvia | [ACT55367](http://www.ncbi.nlm.nih.gov/protein/254034418?ordinalpos=1&itool=EntrezSystem2.PEntrez.Sequence.Sequence_ResultsPanel.Sequence_RVDocSum) | 231/268 | 86 | 1,00E-144 | 387 |
| Contig_90c_3172_4 | Pm shem 3 for shematrin | *Pinctada maxima* | Bivalvia | BAG15913 | 220/245 | 89 | 1,00E-130 | 251 |
| Contig_90c_600_4 | MSI 60 (insoluble protein) | *Pinctada fucata* | Bivalvia | [BAA20466](http://www.ncbi.nlm.nih.gov/protein/2204081?ordinalpos=1&itool=EntrezSystem2.PEntrez.Sequence.Sequence_ResultsPanel.Sequence_RVDocSum) | 256/326 | 78 | 1,00E-123 | 738 |
| Contig_90c_441_3 | Pm shem 1 for shematrin | *Pinctada maxima* | Bivalvia | BAG15911 | 199/230 | 86 | 1,00E-113 | 286 |
| Contig_90c_24_4 | Shematrin-6 | *Pinctada fucata* | Bivalvia | BAE93438 | 207/323 | 64 | 1,00E-103 | 302 |
| Contig_90c_392_2 | Tyrosinase | *Pinctada fucata* | Bivalvia | AAZ66340 | 172/269 | 63 | 1,00E-102 | 445 |
| Contig_90c_707_5 | KRMP 5 | *Pinctada margaritifera* | Bivalvia | ABO87298 | 142/144 | 98 | 7,00E-96 | 144 |
| Contig_90c_117_4 | ferritin-like | *Pinctada fucata* | Bivalvia | [AAQ12076](http://www.ncbi.nlm.nih.gov/protein/33333949?ordinalpos=1&itool=EntrezSystem2.PEntrez.Sequence.Sequence_ResultsPanel.Sequence_RVDocSum) | 169/177 | 95 | 5,00E-95 | 206 |
| Contig_90c_2402_1 | KRMP 9 | *Pinctada margaritifera* | Bivalvia | ABP57447 | 136/137 | 99 | 5,00E-92 | 137 |
| Contig_90c_12451_4 | Perline matrix protein | *Pinctada margaritifera* | Bivalvia | ABG24165 | 140/140 | 100 | 2,00E-89 | 140 |
| Contig_90c_51_2 | mpn88-lack6 | *Pinctada fucata* | Bivalvia | BAH05013 | 249/543 | 45 | 1,00E-88 | 801 |
| Contig_90c_2458_5 | KRMP 10 | *Pinctada margaritifera* | Bivalvia | ABP57448 | 132/139 | 94 | 7,00E-85 | 135 |
| Contig_90c_783_6 | [Calmodulin](http://srs.ebi.ac.uk/srsbin/cgi-bin/wgetz?-e+%5B{EMBL EMBLCON EMBLANN}-ProteinID:ACI22622*%5D) | [*Hyriopsis schlegelii*](http://www.uniprot.org/taxonomy/319528) | Bivalvia | ACI22622 | 148/149 | 99 | 2,00E-80 | 149 |
| Contig_90c_8234_1 | Shematrin-7 | *Pinctada fucata* | Bivalvia | BAE93439 | 192/404 | 47 | 1,00E-78 | 315 |
| Contig_90c_1789_5 | Mantle gene 8 | *Pinctada fucata* | Bivalvia | AAZ76262 | 146/230 | 63 | 2,00E-71 | 223 |
| Contig_90c_581_4 | Shematrin-5 | *Pinctada fucata* | Bivalvia | BAE93437 | 158/288 | 54 | 1,00E-70 | 278 |
| Contig_90c_182_2 | MSI25 (hypothetical protein) | *Pinctada fucata* | Bivalvia | [BAF43717](http://www.ncbi.nlm.nih.gov/protein/121308649?ordinalpos=1&itool=EntrezSystem2.PEntrez.Sequence.Sequence_ResultsPanel.Sequence_RVDocSum) | 134/175 | 76 | 7,00E-70 | 175 |
| Contig_90c_81_3 | KRMP 7 | *Pinctada margaritifera* | Bivalvia | ABP57445 | 114/127 | 89 | 3,00E-69 | 119 |
| Contig_90c_1483_2 | KRMP 8 | *Pinctada margaritifera* | Bivalvia | ABP57446 | 110/140 | 78 | 9,00E-69 | 110 |
| Contig_90c_557_6 | linkine | *Pinctada margaritifera* | Bivalvia | ABO87300 | 111/111 | 100 | 7,00E-64 | 111 |
| Contig_90c_22546_2 | L-type voltage-dependent calcium channel beta subunit | *Pinctada fucata* | Bivalvia | ABL98211 | 114/123 | 92 | 1,00E-62 | 391 |
| Contig_90c_795_6 | Mantle protein 12 | *Pinctada fucata* | Bivalvia | AAZ22321 | 107/163 | 65 | 4,00E-60 | 158 |
| Contig_90c_697_5 | Mantle gene 2 | *Pinctada fucata* | Bivalvia | AAZ76256 | 114/178 | 64 | 6,00E-58 | 190 |
| Contig_90c_11769_5 | neuronal calcium sensor-1 | Lymnaea stagnalis | | Gastropoda | | --- | | [AAZ66779](http://www.ncbi.nlm.nih.gov/protein/161345057?ordinalpos=1&itool=EntrezSystem2.PEntrez.Sequence.Sequence_ResultsPanel.Sequence_RVDocSum) | 98/165 | 59 | 6,00E-56 | 191 |
| Contig_90c_95_3 | Mantle protein 10 | *Pinctada fucata* | Bivalvia | AAZ22319 | 99/175 | 56 | 2,00E-54 | 191 |
| Contig_90c_2066_1 | PMMG1 | *Pinctada maxima* | Bivalvia | ACJ13444 | 100/110 | 90 | 4,00E-54 | 140 |
| Contig_90c_20552_5 | bone morphogenic protein type 2 receptor | *Crassostrea gigas* | Bivalvia | CAD20574 | 93/159 | 58 | 6,00E-53 | 1174 |
| Contig_90c_883_3 | Mantle gene 3 | *Pinctada fucata* | Bivalvia | AAZ76257 | 84/116 | 72 | 7,00E-51 | 121 |
| Contig_90c_1652_1 | BMP2/4 | *Saccostrea kegaki* | Bivalvia | BAG68618 | 128/439 | 29 | 2,00E-43 | 421 |
| Contig_90c_334_6 | Prismalin-14 | *Pinctada fucata* | Bivalvia | BAD27406 | 91/133 | 68 | 1,00E-41 | 121 |
| Contig_90c_3816_2 | Prisilkin-39 | *Pinctada fucata* | Bivalvia | ACJ06766 | 79/124 | 63 | 7,00E-41 | 406 |
| Contig_90c_275_3 | Mantle protein 9 | *Pinctada fucata* | Bivalvia | AAZ22318 | 87/171 | 50 | 2,00E-40 | 209 |
| Contig_90c_5412_4 | G-protein a-subunit s class | *Pinctada fucata* | Bivalvia | Q6TP31 | 97/216 | 44 | 4,00E-40 | 377 |
| Contig_90c_7122_3 | metallothionein mRNA | *Pinctada maxima* | Bivalvia | ACJ22893 | 61/75 | 81 | 1,00E-39 | 75 |
| Contig_90c_299_1 | Carbonic anhydrase precursor | *Tridacna gigas* | Bivalvia | AAX16122 | 92/259 | 35 | 3,00E-37 | 600 |
| 132505_2332_2165_6 | Sarco/endoplasmic reticulum calcium ATPase isoform A | *Pinctada fucata* | Bivalvia | [ABS19815](http://www.ncbi.nlm.nih.gov/protein/152003983?ordinalpos=1&itool=EntrezSystem2.PEntrez.Sequence.Sequence_ResultsPanel.Sequence_RVDocSum) | 76/79 | 96 | 4,00E-37 | 1007 |
| Contig_90c_17445_2 | calcium-dependent protein kinase | *Crassostrea gigas* | Bivalvia | [AAU93878](http://www.ncbi.nlm.nih.gov/protein/53801337?ordinalpos=1&itool=EntrezSystem2.PEntrez.Sequence.Sequence_ResultsPanel.Sequence_RVDocSum) | 73/106 | 68 | 5,00E-37 | 248 |
| Contig_90c_19664_1 | Shematrin-4 | *Pinctada fucata* | Bivalvia | BAE93436 | 81/134 | 60 | 6,00E-37 | 306 |
| 393137_2638_0208_3 | Plasma membrane calcium ATPase | *Pinctada fucata* | Bivalvia | ABL63470 | 75/86 | 87 | 1,00E-36 | 1189 |
| Contig_90c_2165_6 | calcium-ATPase | *Mizuhopecten yessoensis* | Bivalvia | [BAA37143](http://www.ncbi.nlm.nih.gov/protein/4165020?ordinalpos=1&itool=EntrezSystem2.PEntrez.Sequence.Sequence_ResultsPanel.Sequence_RVDocSum) | 74/79 | 93 | 3,00E-36 | 993 |
| Contig_90c_928_2 | Putative uncharacterized protein F18 | *Crassostrea nippona* | Bivalvia | BAG50305 | 79/144 | 54 | 9,00E-35 | 157 |
| Contig_90c_666_1 | calcium/calmodulin-dependent serine protein kinase 1 | *Lymnaea stagnalis* | Gastropoda | [AAO83853](http://www.ncbi.nlm.nih.gov/protein/29378343?ordinalpos=1&itool=EntrezSystem2.PEntrez.Sequence.Sequence_ResultsPanel.Sequence_RVDocSum) | 87/274 | 31 | 1,00E-33 | 915 |
| Contig_90c_5769_1 | KRMP 2 | *Pinctada fucata* | Bivalvia | AAZ95764 | 61/98 | 62 | 2,00E-33 | 101 |
| Contig_90c_22805_2 | voltage-dependent L-type calcium channel alpha-1 subunit isoform c | *Lymnaea stagnalis* | Gastropoda | [AAO83840](http://www.ncbi.nlm.nih.gov/protein/29378317?ordinalpos=1&itool=EntrezSystem2.PEntrez.Sequence.Sequence_ResultsPanel.Sequence_RVDocSum) | 70/102 | 68 | 2,00E-30 | 538 |
| Contig_90c_19560_2 | Mantle gene 4 | *Pinctada fucata* | Bivalvia | AAZ76258 | 56/92 | 60 | 1,00E-27 | 158 |
| Contig_90c_3516_1 | sarcoplasmic calcium-binding protein | *Mizuhopecten yessoensis* | Bivalvia | [BAA89420](http://www.ncbi.nlm.nih.gov/protein/6692814?ordinalpos=1&itool=EntrezSystem2.PEntrez.Sequence.Sequence_ResultsPanel.Sequence_RVDocSum) | 55/85 | 64 | 2,00E-27 | 178 |
| Contig_90c_8263_6 | Jacalin-related lectin PPL2-a | *Pteria penguin* | Bivalvia | BAG80527 | 51/133 | 38 | 1,00E-25 | 168 |
| Contig_90c_3996_3 | hypothetical protein (pfp-16) | *Pinctada fucata* | Bivalvia | BAF43718 | 73/146 | 50 | 4,00E-25 | 137 |
| Contig_90c_12029_4 | N19 (Nacre Protein) | *Pinctada fucata* | Bivalvia | [BAF76073](http://www.ncbi.nlm.nih.gov/protein/156622557?ordinalpos=1&itool=EntrezSystem2.PEntrez.Sequence.Sequence_ResultsPanel.Sequence_RVDocSum) | 53/105 | 50 | 9,00E-25 | 197 |
| 292259_2670_2456_1 | Aspein (shell matrix protein) | *Pinctada fucata* | Bivalvia | BAD00044 | 61/70 | 87 | 2,00E-23 | 413 |
| 268637_2368_1695_5 | Amorphous calcium carbonate binding protein 1 (ACCBP) | *Pinctada fucata* | Bivalvia | [ABF13208](http://www.ncbi.nlm.nih.gov/entrez/viewer.fcgi?db=nuccore&val=93359256) | 45/79 | 56 | 8,00E-23 | 240 |
| Contig_90c_1350_1 | MSI7 (glycine-rich shell matrix protein) | *Pinctada fucata* | Bivalvia | [AAQ08227](http://www.ncbi.nlm.nih.gov/protein/33325246?ordinalpos=1&itool=EntrezSystem2.PEntrez.Sequence.Sequence_ResultsPanel.Sequence_RVDocSum) | 47/75 | 62 | 2,00E-19 | 95 |
| Contig_90c_5271_4 | homeobox protein 4 | *Gibbula varia* | Gastropoda | ACX84672 | 45/77 | 58 | 7,00E-19 | 157 |
| 207877_3232_2965_5 | lectin | *Pteria penguin* | Bivalvia | [BAB03232](http://www.ncbi.nlm.nih.gov/protein/9309336?ordinalpos=1&itool=EntrezSystem2.PEntrez.Sequence.Sequence_ResultsPanel.Sequence_RVDocSum) | 38/79 | 48 | 2,00E-14 | 183 |
| Contig_90c_1569_6 | engrailed | *Saccostrea kegaki* | Bivalvia | BAG68617 | 43/113 | 38 | 7,00E-14 | 229 |
| 172458_3474_2721_5 | C-type lectin 1 | *Pinctada fucata* | Bivalvia | ACO36045 | 39/82 | 47 | 4,00E-13 | 287 |
| Contig_90c_12263_2 | engrailed | *Haliotis asinina* | Gastropoda | ABC00198 | 32/63 | 50 | 5,00E-12 | 222 |
| 298867_2192_0444_6 | Incilarin C | *Incilaria fruhstorferi* | Gastropoda | BAA19863 | 34/82 | 41 | 5,00E-12 | 156 |
| Contig_90c_86_6 | Perlwapin | *Haliotis laevigata* | Gastropoda | P84811 | 40/107 | 37 | 2,00E-11 | 134 |
| Contig_90c_1540_3 | Mantle protein 11 | | *Pinctada fucata* | | --- | | Bivalvia | AAZ22320 | 60/186 | 32 | 2,00E-11 | 199 |
| Contig_90c_2046_6 | Incilarin A | *Incilaria fruhstorferi* | Gastropoda | BAA19861 | 30/94 | 31 | 1,00E-10 | 150 |
| Contig_90c_4783_1 | Veliger mantle 1 | *Haliotis asinina* | Gastropoda | ABD47938 | 43/155 | 27 | 5,00E-09 | 245 |
| 431794_3530_0210_4 | Perlucin | *Haliotis laevigata* | Gastropoda | P82596 | 27/71 | 38 | 9,00E-09 | 155 |
| Contig_90c_26454_2 | Prismin_2 | *Pinctada fucata* | Bivalvia | BAG28185 | 26/46 | 56 | 1,00E-08 | 51 |
| Contig_90c_7875_3 | EP protein precursor | *Mytilus edulis* | Bivalvia | AAQ63463 | 37/129 | 28 | 3,00E-08 | 236 |
| 141091_3618_1772_3 | Perlucin-7 | *Haliotis discus discus* | Gastropoda | ABO26596 | 28/82 | 31 | 4,00E-08 | 162 |
| Contig_90c_554_6 | Asprich 1 | *Atrina rigida* | Bivalvia | AAU04814 | 35/91 | 38 | 2,00E-06 | 161 |
| Contig_90c_5311_4 | Perlustrin | *Haliotis laevigata* | Gastropoda | P82595 | 25/64 | 39 | 7,00E-06 | 84 |
| Contig_90c_3116_2 | C-type lectin 2 | *Pinctada fucata* | Bivalvia | ACO36046 | 38/154 | 24 | 3,00E-05 | 168 |
| Contig_90c_20640_3 | hypothetical protein MPP1 | *Mercenaria mercenaria* | Bivalvia | [BAF43714](http://www.ncbi.nlm.nih.gov/protein/121308643?ordinalpos=1&itool=EntrezSystem2.PEntrez.Sequence.Sequence_ResultsPanel.Sequence_RVDocSum) | 14/31 | 45 | 2,00E-04 | 106 |
| 129509_3511_1612_1 | TFG beta signaling pathway factor | *Pinctada fucata* | Bivalvia | ABX57736 | 21/52 | 40 | 4,00E-04 | 413 |
| Contig_90c_18304_5 | Calmodulin | *Conus cuneolus* | Gastropoda | AAS01355 | 17/17 | 100 | 0.001 | 17 |
